# Anti-mycobacteria potential and synergistic effects of combined crude extracts of selected medicinal plants used by Bapedi traditional healers to treat tuberculosis related symptoms in Limpopo Province, South Africa

**DOI:** 10.1186/s12906-016-1521-2

**Published:** 2017-02-24

**Authors:** Nancy Patience Motlalepula Komape, Victor Patrick Bagla, Prudence Kabongo-Kayoka, Peter Masoko

**Affiliations:** 1 Department of Biochemistry, Microbiology and Biotechnology, Faculty of Science and Agriculture, University of Limpopo, Private Bag x1106, , Sovenga, 0727 South Africa; 2 Phytomedicine Programme, Department of Paraclinical Sciences, Faculty of Veterinary Sciences, University of Pretoria, Private Bag x04, Onderstepoort, 0110 South Africa

**Keywords:** *Combretum heroroense*, *Citrus lemon*, *Apodytes dimidiata*, Minimum inhibitory concentration, Bio-autography, Anti-oxidant activity, Multi-drug resistant tuberculosis strain

## Abstract

**Background:**

Tuberculosis is an infectious communicable disease and the causative agent of the disease has over the years developed resistance to streamline chemotherapeutic agents with dire consequences and there is a need for development of new and more potent alternatives.

**Methods:**

Constituents of leaves material of *Combretum heroroense*, *Citrus lemon* and *Apodytes dimidiata* were serially extracted using solvents of varying polarity. TLC finger print profile of the different extracts were determined by spraying eluted plates with vanillin sulphuric acid and 2, 2- diphenylpicryl hydrazyl (DPPH) for the presence of antioxidant constituents. Presence of different phytochemicals was determined using standard chemical test. Bioautography was used to determine the number of compounds present in sub-fractions active against *Mycobacterium smegmatis*. Minimum inhibitory concentration (MIC) values extract and sub-fractions were determined using serial microplate dilution method against *M. smegmatis* (ATCC 1441), *M. tuberculosis* (ATCC H37Rv) and multi-drug resistant TB (MDR-TB) field strain. Synergy of the crude extracts of the three plants was determined using microplate dilution method against *M. smegmatis*.

**Results:**

Mass extracted by different solvents was less than 6% dry weight for all the plants. Phlobatannins were not detected in *A. dimidiata*, *C. heroroense* and *C. lemon* as well as cardiac glycosides in *C. lemon* and *A. dimidiata*, and saponins in *C. heroroense*. Sub-fractions of the different plants were shown to contain constituents with antioxidant activity with the highest number detected in *C. heroroense*. Bioautography results reveal the presence of a compound(s) in the ethyle acetate sub-fraction of *C. heroroense* and butanol, methanol/water, ethyl acetate and water no.2 subfractions of *A. dimidiata*, active against *M. smegmatis* that were not shown to have antioxidant capacity. MIC results for different crude extracts of the three plants against *M. smegmatis* ranges from 0.1 to 3 mg/ml. The average MIC for the synergistic effect of the plants ranged from 0.04 mg/ml to 1.25 mg/ml. An activity greater than that obtained for the reference drugs was shown for the butanol and hexane fractions of *A. dimidiata* (0.47 mg/ml) against the field strain of MDR-TB while that obtained for the *M.TB* (ATCC H37Rv) was 0.31 mg/ml.

**Conclusion:**

A significant finding shown in this study reveals the potent anti-mycobacteria potential of sub-fractions of *A. dimidiata* against MDR-TB field strain that can lead to the isolation of compounds that can be used to counter resistant strains of tuberculosis.

## Background

Tuberculosis (TB) remains one of the major causes of death among infectious diseases and has become a global public health threat. Globally about 5% of the mycobacterium causing TB has developed resistance (multidrug-resistant TB (MDR-TB) to streamline therapeutic agents in 2014. Drug resistance surveillance data also show an estimate of 480, 000 people that have developed MDR-TB within this period, with about 190, 000 deaths as a result of MDR-TB [1]. South Africa is among the 27 high burden listed countries affected by MDR-TB and accounts for 390,000 patients representing 1.8% of global statistic [1]. MDR-TB develops when the tuberculosis bacterial that a person is infected with, are resistant to at least two of the most important TB drugs (isoniazid (INH) and rifampicin (RMP). In light of the development of resistance in those infectious diseases with existing drugs, one strategy employed in traditional herbal medicine to overcome this phenomenon is the combination of herbal remedies. To this effect, some authors have attempted the combination of antibiotics with plant extract [2] while others have focused on plant extract combinations to achieve a more potent antimicrobial activity [3].

Herbal medicine represents one of the most important fields of traditional medicine all over the world and can offer hope for the development of alternate medicines for the treatment of tuberculosis. To promote proper use of herbal medicine and determine their potential as a source of new drugs, it is essential to study medicinal plants which have folklore reputation in more intensified way [4, 5]. The main objective of this study was to determine the anti-mycobacterial activity of the leaf extracts of three selected plants used by Bapedi traditional healer namely, *Citrus lemon*, *Combretum heroroense* and *Apodytes dimidiata* with the traditional indication of treatment of tuberculosis related symptoms such as persistent coughing, weight loss, sweating at night and blood in the sputum. The effects of the combination of the different extracts of the plants were also access for possible synergistic effect against *M. smegmatis. A. dimidiata* that exhibited the most potent activity was sub-fractionated into the, butanol, hexane, ethyl acetate, methanol and water fractions 1 and 2 and activities tested on a MDR-TB field strain. We report for the first time potent anti-mycobateria activity of sub-fractions of *A. dimidiata* against a MDR-TB field strain and the Mycobacteria tuberculosis (ATCC H37Rv) strain. The phytochemical constituents of extracts of the selected plants were also assessed.

## Methods

### Plant collection and storage

The leaves of *C. lemon* (UNIN 12330) were collected from the University of Limpopo Campus while those of C. *heroroense* (LNBG 1977/71) and *A. dimidiata* (LNBG1969/46) were collected from Lowveld National Botanical Garden Nelspruit, South Africa. C. *heroroense* and *A. dimidiata* voucher specimens in the garden herbarium and tree labels verified the identity of the plants. Plants were confirmed by Mr Willem Froneman (Control Horticulturist). He also provided plants accession details. *C. lemon* leaves voucher specimens in the garden herbarium and tree labels verified the identity of the plant. Plant was confirmed by Dr Bronwyn Egan (Herbarium).

The plants were collected based on their ethnopharmacological information provided by traditional healers in the Sekhukhune, Waterberg and Capricorn District of Limpopo Province. The leaves of the plants were air dried at room temperature for two weeks and milled into fine powder using a blender and stored in dark glass bottles until use.

### Extraction procedure

Leaf powder (1 g) of the plants were each extracted using 10 ml of solvents of varying polarities, namely; hexane, dichloromethane, methanol and acetone in 50 ml centrifuge tubes. The mixtures were exhaustively extracted and filtered into pre-weighed labelled vails using Whatman no. 4 filter paper. The solvent was removed under a stream of air at room temperature.

### Phytochemical analysis

The phytochemical constituents of the plant extracts were analysed by thin layer chromatography (TLC) using aluminium-backed TLC plates (Merck, silica gel 60 F254) according to the method of Kotze and Eloff [6]. The TLC plates were developed under saturated conditions with each of the three mobile phases differing in polarity viz. ethyl acetate: methanol: water (40:5.4:4), [EMW] (polar/neutral); chloroform: ethyl acetate: formic acid (6:4:1), [CEF] (intermediate polarity/acidic); and benzene: ethanol: ammonia hydroxide (9:1:0.1): [BEA] (non-polar/basic). The separated compounds on the chromatograms were visualized under ultraviolet (UV) light (254 and 360 nm) sprayed with vanillin-sulphuric acid and heated at 110 °C for colour development.

### Determination of antioxidant activity

The potential antioxidant activity of the plant extracts was determined on the basis of the scavenging activity of stable 1,1- diphenyl- 2-picrylhydrazyl (DPPH). The chromatograms were prepared as above, in EMW, CEF, and BEA solvent systems. The chromatograms were sprayed with 0.2% DPPH to visualize any potential antioxidant compounds within the separated plant extracts [7].

### Test for the presence of various phytochemicals

The extracts of the three plants that were extracted with acetone were tested for the presence of saponins, phlobatannins, tannins, terpenes/terpenoids, cardiac glycosides, flavonoids and steroids using standard procedures as described by Borokini and Omotayo [8].

### Alkaloids

Method described by Harbone [9] was adopted using Drangendoff’s reagent. About 0.2 g of finely grounded leaves were extracted using 95% ethanol and solvent evaporated to dryness. The extracts were re-dissolved in 5 ml of 1% HCl and 5 drops of Drangendoff’s reagent added. A colour change (orange to orange red precipitate) was observed to draw inference.

### Saponin

The method of Odebiyi and Sofowora [10] persistent frothing test for saponin was used. Thirty millilitres of water was added to 1 g powdered leaf sample. The mixture was vigorously shaken and heated. The samples were observed for the persistence appearance of foam lasting for at least 15 min confirmed the presence of saponins.

### Phlobatannin

Powdered leaf sample (0.2 g) was dissolved in distilled water and filtered. The filtrate was then boiled in 2% HCl solution. The deposition of a red precipitate confirmed the presence of phlobatannins [11].

### Tannins

The method of Trease and Evans [12] was adopted. Powdered leaf (0.5 g) samples were dissolved in 5 ml of distilled water, boiled gently and cooled. To 1 ml of each extract, 3 drops of ferric chloride solution was added. The formation of green coloured precipitate indicates the presence of tannins.

### Terpenes/terpenoids

The Salkowski test was used to test for the presence of terpenes/terpenoids in the different extracts. Five millilitres of powdered leaf sample was mixed in 2 ml chloroform and 3 ml concentrated sulphuric acid was then carefully added to form a layer. The development of greyish colour indicates the presence of terpenes/terpenoids [13].

### Steroids

Acetic anhydride (2 ml) was added to 0.5 g powdered leaf material of each plant sample, followed by 2 ml sulphuric acid. Colour changes from violet to blue or green in some plants indicates the presence of steroids [14].

### Cardiac glycosides

The Keller-Killani test was employed. Methanolic plant extracts (5 ml) of all the plants studied were treated with 2 ml of glacial acetic acid, containing one drop of ferric chloride solution. This was underplayed with 1 ml of concentrated sulphuric acid. Brown ring was formed at the interface which indicated the presence of deoxysugar cardenoloides. A violet ring would appear beneath the brown ring, while in the acetic acid layer, a greenish ring may also form gradually throughout the layer.

### Flavonoids

Diluted ammonia solution (5 ml) was added to a portion of the aqueous filtrate of each plant extract, followed by addition of concentrated sulphuric acid. The formation of a yellow precipitate indicated the presence of flavonoids [8].

### Solvent- solvent fractionation

**Fig. 1:**
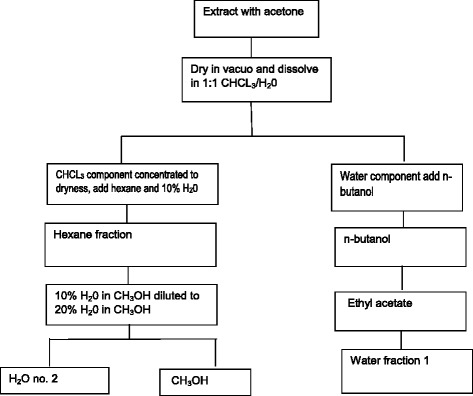
Schematic representation of solvent-solvent fractionation method

The solvent-solvent fractionation method was used to separate the fractions of different extracts. Acetone was used as the extraction solvent because intermediate polarity capable of extraction both polar and non-polar constituents. The procedure as outlined by the USA National Cancer Institute [15] was followed with minor modifications. The extracts were fractionated using solvents of different polarities as indicated in the flow chart below (Fig. 1).

### Test Organism

The test organism, *Mycobacterium smegmatis* (ATCC1441), was obtained from School of Molecular and Cell Biology, University of the Witwatersrand. The test organism was maintained and grown in Middlebrook 7H9 (Fluka M0178) broth with glycerol (Fluka 49769) or Tween 80 (Fluka 93780) and Middlebrook Oleic Albumin Dextrose Catalase (OADC) growth supplement (Fluka M0553).

### Mycobacterium tuberculosis multidrug resistant isolates (MDR-TB)

A clinical isolate of multidrug resistant *Mycobacterium tuberculosis* (MDR-TB) was used. The isolate was obtained from patients admitted to the MDR-TB ward at Tshepong hospital in Klerksdorp, North West Province of South Africa in December 2012. Samples of sputum were submitted to the National Health Laboratory Services (NHLS) in Pretoria for culture in liquid medium and PCR/Line Probe Assay. The isolate was found to be resistant to isoniazid and rifampicin.

### Rapidly growing mycobacteria

The *Mycobacterium tuberculosis* H37Rv which is routinely used as reference strain at NHLS was obtained from the American Type Culture Collection (ATCC) number 25177.

### Maintenance of cultures of pathogenic isolate

Fresh culture was used in the relevant assays. The pathogenic isolate of *Mycobacterium* spp. kept at room temperature on Lowenstein-Jensen (LJ) slants supplemented with glycerol was used within a month. Prior to each assay, culture was revived in liquid medium, Middlebrook 7H9, using MGIT 960 tubes which was incubated at 37 °C in the BACTEC MGIT 960 instrument, in which it was automatically monitored each hour for fluorescence development for 42 days or until a positive signal developed. Bacterial suspensions from MGIT tubes were then subcultured on solid medium LJ slants with glycerol for *M. tuberculosis*. Löwenstein Jensen tubes were then incubated in a walk-in incubator at 37 °C for 4 to 6 weeks. A stained Ziehl Neelsen smear was made from the sediment of the MGIT tube and the slant of LJ medium. Reference culture of *M. tuberculosis* H37Rv (ATCC 25177) was used as positive controls.

### Bioautography assay using *Mycobacterium smegmatis*

Bio-autography assays was carried out on TLC plates according to Beque and Kline [16] to detect the main bioactive compounds within the crude extracts. TLC plates were loaded with 10 μl of 10 mg/ml solution of each extract as described under phytochemical analysis. Chromatograms were left to fan-dry for three days to completely evaporate the eluent solvents and sprayed with *M. smegmatis*. Sprayed chromatograms were then incubated at 37 °C for 24 h in humid conditions. After incubation the bioautograms were sprayed with visualization stain (iodonitrotetrazolium salt), and incubated further at 37 °C for 24 h in closed containers to allow for colour development. The appearances of a clear zones/white spots on the bioautograms were considered as areas of inhibition of growth whereas pink-red colour indicated bacterial growth.

### Minimum Inhibitory Concentration (MIC) determination using *M. smegmatis*

Minimum Inhibitory Concentration values were determined using the serial micro-dilution method described by Eloff [17]. The MIC is described as the lowest concentration of the compounds inhibiting the growth of the microorganisms. Dried extracts were re-dissolved in acetone to a concentration of 10 mg/ml. The test was carried out in triplicates. The plant extracts were combined 50 μl each and the final mixture was 100 μl. The mixture of combined plant extracts were then serially diluted 50% with water in a 96-well microtitre plates. Bacterial cultures were sub-cultured and transferred into fresh Middlebrook 7H9 broth and 100 μl of the culture was transferred into each well. Acetone blanks were included and rifampicin was used as a positive control. The microtitre plate was incubated at 37 °C for 24 h. After incubation, 20 μl of ρ- iodonitrotetrazolium violet (Sigma) (INT) dissolved in water was added to each of microplates wells as an indicator for growth. The plates were covered and further incubated for 30 min at 37 °C and 100% relative humidity for colour development. Purple- red colour indicates microbial growth and clear wells indicate inhibition of microbial growth by extracts.

### Minimum inhibitory concentration (MIC) determination using pathogenic strain

The MIC values were determined using the serial microplate method developed by [17], slightly modified for mycobacteria by [18]. Mycobacterial suspensions were prepared from a pure culture of fresh colonies from solid medium and suspended in Middlebrook 7H9 (M7H9) liquid medium supplemented with 10% OADC. These colonies were transferred into a sterile screw capped tube containing 3 ml of M7H9 broth and homogenized by placing the tube on a Vortex mixer for 5 min. After the larger particles had settled, the mycobacterial suspension was adjusted to McFarland no.1 turbidity standard by adding more broth [19].

The assay was performed using sterile 96-well microplates with round bottoms. The sample to be tested was prepared at a concentration of 10 mg/ml prior to serial dilution. One hundred μl of M7H9 broth was added to all the wells from column 1 to 12 and then 100 μl of the sample to be tested were added to the relevant wells in the first row. A two fold serial dilution was carried out leaving 100 μl of different concentrations of diluted tested samples in each well starting with a concentration of 2.5 mg/ml in the first wells. Then 100 μl of the relevant bacterial suspension were added to the relevant wells. Each test was triplicated (3 wells). Tested samples also included acetone, pure broth as negative control and reference drug isoniazid including rifampicin and streptomycin as positive controls starting with a concentration of 100 μg/ml. The microplates were covered and sealed in plastic bags, placed in humid chambers to minimize the evaporation of the culture medium and incubated at 37 °C for a period of 7 to 15 days.

At the end of incubation, a volume of 40 μl of 0.2 mg/ml of iodonitrotetrazolium chloride (INT) was added to each well, plates were incubated for 30 min or longer at 37 °C and the development of colour observed. A coloured red-purple formazan or pink color indicated the reduction of INT by metabolizing organisms whereas a yellow color or decrease in color indicated the inhibition of bacterial growth [17]. If the colour development was not strong enough for slow growing organisms, plates were incubated much longer and monitored.

## Results

### Mass extracted from the plants using different solvents

Extraction was done as an initial step towards extraction of active constituents contained in the selected plants. Leaf material of *C. heroroense*, *C. lemon* and *A. dimidiata* were extracted using hexane, dichloromethane, methanol and acetone. Table 1 represents the percentage mass extracted from 1 g of plant material using the different solvents employed.

**Table 1:** Percentage mass extracted (g) from hexane, dichloromethane, methanol and acetone of *C. heroroense*, *C. lemon* and *A. dimidiata*

		Plant species		
Extracts	*C. lemon*	*C.heroroense*	*A. dimidiata*	Average
Hexane	1.73	0.01	1.37	1.04
Dichloromethane	0.29	0.3	5.38	1.99
Methanol	2.62	5.33	4.52	4.16
Acetone	0.34	3.35	0.46	1.38
Total yield	4.98	8.99	11.73	8.57

Chloroform extract of *A. dimidiata* had the highest total yield of 11.73%, followed by *C.heroroense* (8.99%) and then *C. lemon* (4.98%). For all the plants, methanol had the average percentage yield (4.16%) as compared to the other extracts and hexane had the lowest percentage yield (1.04%). This indicates that in all the plants, more polar compounds were extracted as compared to the non- polar compounds.

### Thin layer chromatography (TLC) finger print profile of the plants

**Fig. 2:**
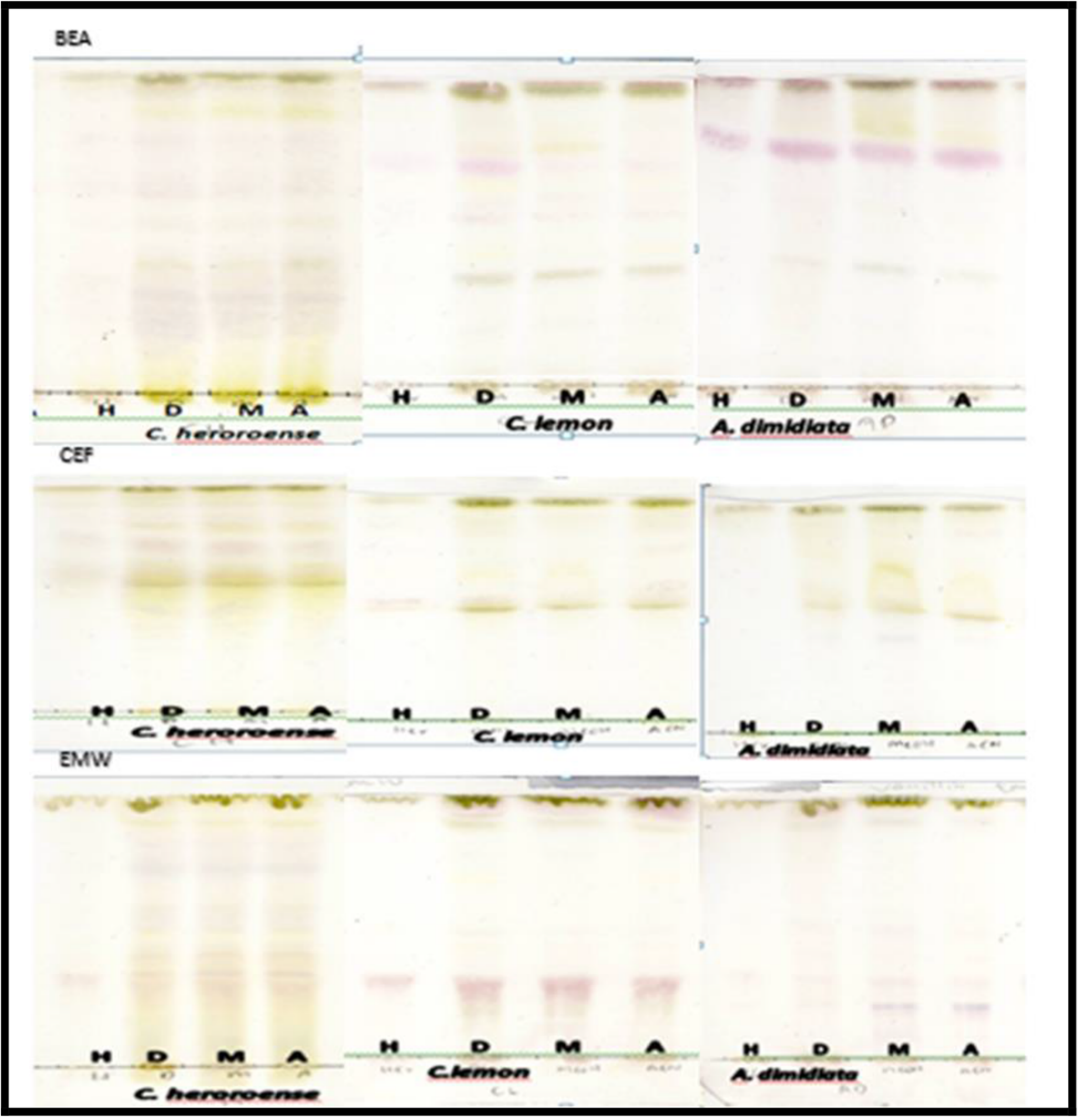
Thin layer chromatography profiling of *Combretum heroroense*, *Citrus lemon* and *A. dimidiata* of the plates developed in BEA (top), CEF (middle) and EMW (bottom) sprayed with vanillin sulphuric acid

Phytochemical analysis of the crude extracts was conducted using thin layer chromatography (TLC). In Fig. 2, the TLC plates were developed in BEA, CEF and EMW respectively, to illustrate the finger print profile of extracts of the selected plants. Different constituents depending on the type of extract were observed on TLC. Constituents in all the plants were best separated in the BEA eluent system when compared to those in the more polar eluent system (EMW).

### Presence of phytochemicals

Phytochemical analysis of the plants showed that tannins, terpenes/terpenoids, steroids and flavonoids were present in all the plants while were phlobatannins absent. *A. dimidiata* and *C. heroroense* had 5 out of the 7 phytochemicals investigated, whereas *C. lemon* had 4 out 7 phytochemicals investigated (Table 2).

**Table 2:** Type of phytochemicals detected in the different plants

Plant sp.	Saponins	Phlobatannins	Tannins	Terpenes/terpenoids	Steroids	Cardiac glycosides	Flavanoids
*A.dimidiata*	+	-	+	+	+	-	+
*C.heroroense*	-	-	+	+	+	+	+
*C.lemon*	-	-	+	+	+	-	+

- = Absence, + = present

### Minimum inhibitory concentration (MIC) and total activity values of plant extracts against *M. smegmatis*

The MIC and total activity values of extracts of selected plants against *M. smegmatis* is presented in Table 3. The dichloromethane extract of *A. dimidiata* showed good activity against the tested pathogen at MIC value of 0.1 mg/ml, followed by the dichloromethane and methanol extracts of *C. lemon* with MIC values of 0.3 mg/ml. Less potent activity was obtained with the hexane and acetone extract of *C. lemon* (MIC 3 mg/ml and 1.3 mg/ml respectively), the hexane and methanol extracts of *C. hororoense* (MIC 1.6 mg/ml and 1.3 mg/ml respectively) and the hexane and acetone extract of *A. dimidiata* (MIC 1.3 mg/ml).

**Table 3:** Minimum inhibitory concentration (MIC) in mg/ml and total activity (ml^−1^) of *Citrus lemon*, *Combretum heroroense* and *Apodytes dimidiata* using hexane, dichloromethane (DCM), methanol and acetone as the extracting solvents

Plant species												
	*C. lemon*				*C. hororoense*				*A. dimidiata*			
	H	D	A	M	H	D	A	M	H	D	A	M
MIC (mg/ml)	3	0.3	1.3	0.3	1.6	0.6	0.6	1.3	1.3	0.1	1.3	0.6
Total activity (ml-1)	7	10	2	87	0.8	5	57	41	11	675	4	75

Rifampicin = 0.13 mg/mL

H-Hexane, D- Dichloromethane, M- Methanol and A- Acetone

Total activity value of an extract or fraction give an indication of the efficacy at which active constituents present in one gram can be diluted and still inhibit the growth of test organisms is shown to be highest for the dichloromethane extract of *A. dimidiata*, followed by the methanol extract of *C. lemon* and *C.hororoense* respectively.

### Test for synergistic effect

Same and different extracts of the selected plants were combined to test for their synesgistic effect against *M. smegmatis* (Table 4). The combination of different extracts of the plants viz the hexane and acetone, and the dichloromethane and methanol extracts of *C. heroroense* and *A. dimidiata* showed excellent activity with MIC value of 0.04 mg/ml, followed by the acetone and methanol, and hexane and methanol extracts of the same plants (MIC 0.08 mg/ml). A similar MIC value (0.08 mg/ml) was also obtained for the combination of the dichloromethane and acetone extracts of *C. lemon* and *C. heroroense*. With same extracts of the different plants, potent activity was shown by the combination of the acetone-acetone and dichloromethane- dichloromethane extracts of *C. heroroense* and *A. dimidiata* (MIC 0.08 mg/ml). Moderate activity was ranging from MIC 0.1.2 to 0.63 mg/ml was shown with the different and same combination of extracts of the selected plants while the lowest activity was obtained at MIC 1.25 mg/ml for the hexane and dichloromethane extracts combination of *C. heroroense* and *A. dimidiata*.

**Table 4:** Minimum inhibitory concentration (MIC) in mg/ml of the combined crude extracts of *Citrus lemon* (CL), *Combretum heroroense* (CH) and *Apodytes dimidiata* (AD) against *M. smegmatis* to show the synergistic effects of the 3 plants

Extracts	CL + CH	CH + AD	AD + CL
H + H	0.31	0.12	0.18
D + D	0.63	0.08	0.63
M + M	0.16	0.12	0.12
A + A	0.16	0.08	0.12
H + D	0.16	1.25	0.63
D + A	0.08	0.16	0.31
A + M	0.16	0.08	0.31
M + H	0.16	0.08	0.63
H + A	0.16	0.04	0.63
D + M	0.16	0.04	0.16

Rifampicin = 0.13 mg/mL

*H* Hexane; *D* Dichloromethane; *M* Methano; *A* Acetone; *CH C.heroroense*, CL = *C.lemon*, *AD A. dimidiata*

### Qualitative antioxidant activity and bioautography of solvent- solvent fractions tested against *M. smegmatis*

**Fig. 3:**
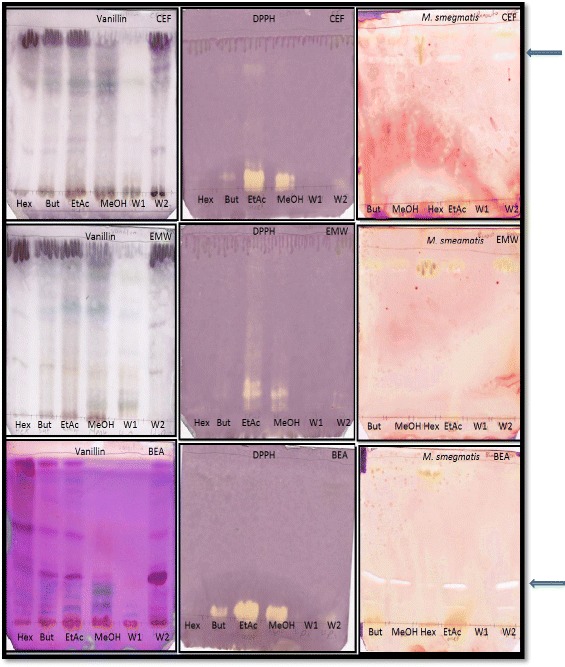
Chromatograms of solvent- solvent fractions [Hex (hexane), But (butanol), EtAc (Ethylacetate), MeOH (methanol), W1 (water fraction no. 1) and W2 (water fraction no. 2)] of *A. dimidiata* developed in CEF (top row), EMW (middle row) and BEA (bottom row) and sprayed with vanillin (left), 1, 2-diphenylpicryl hydrazyl (middle) and *Mycobacterium smegmatis* (right)

The selected plants were solvent-solvent fractionated into the butanol, hexane, ethyl acetate, methanol and water fractions no.1 and 2. The fractions of each of the selected plants were evaluated for the presence of antioxidant and antimycobacterial constituents on TLC. A representative bioautogram depicting fingerprint profile, antioxidant and antimycobacterial activity of *A. dimidiata* is shown in Fig. 3. All the fractions for the three plants namely *A. dimidiata*, *C. heroroense* and *C. lemon* contained constituents with different colours indicating the presence of variety of compounds.

**Fig. 4:**
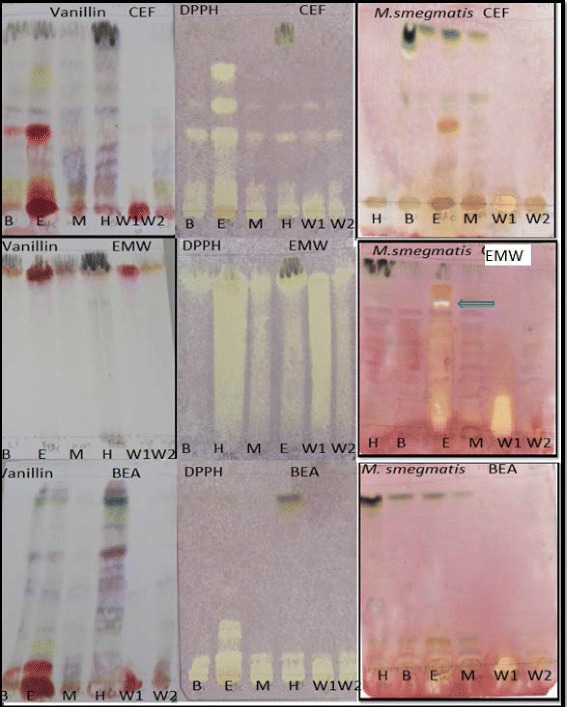
Chromatograms of solvent- solvent fractions [H (hexane), B (butanol), E (Ethylacetate), M (methanol), W1 (water fraction no. 1) and W2 (water fraction no. 2)] of *C. heroroense* developed in CEF (top row), EMW (middle row) and BEA (bottom row) and sprayed with vanillin (left), 1, 2-diphenylpicryl hydrazyl (middle) and *Mycobacterium smegmatis* (right)

**Fig. 5:**
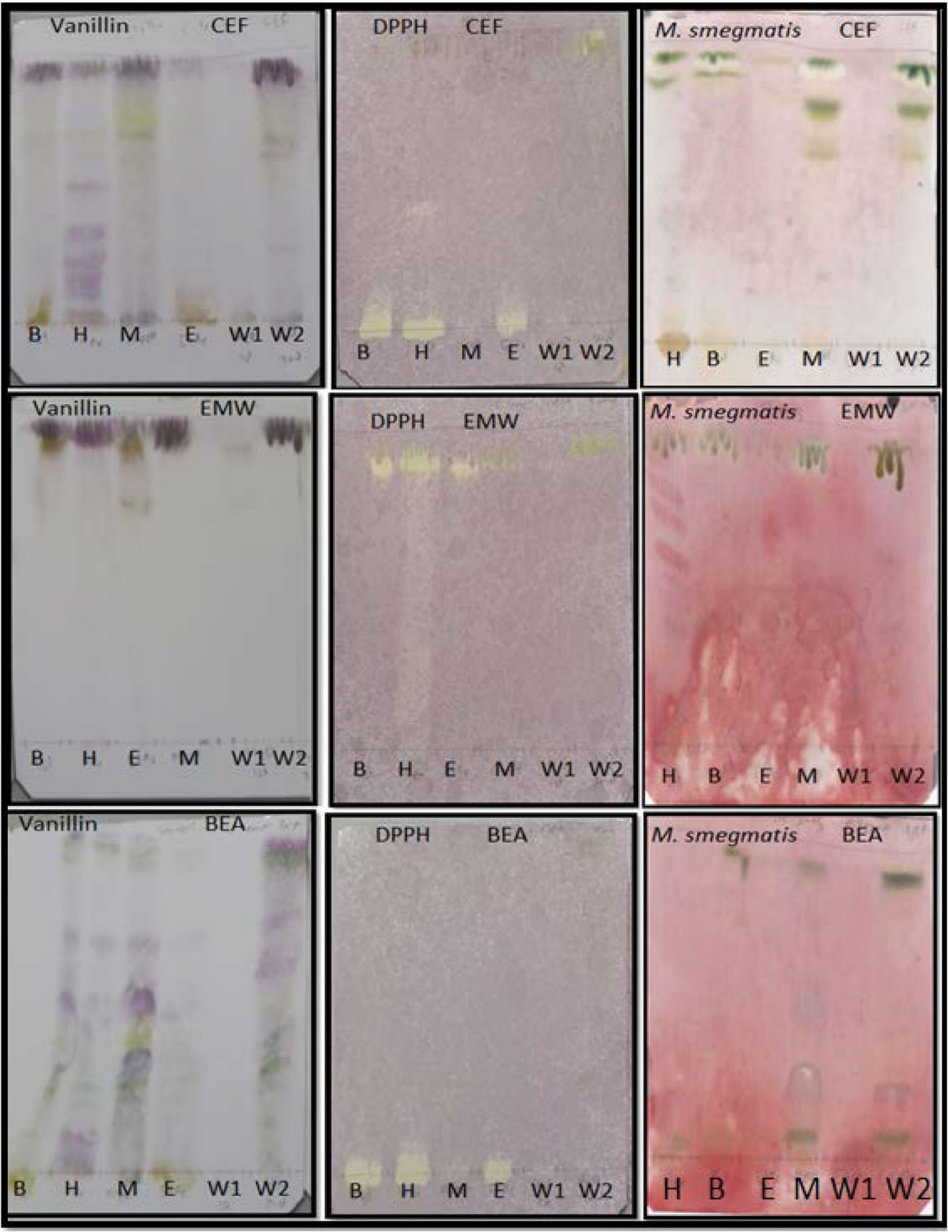
Chromatograms of solvent- solvent fractions [H (hexane), B (butanol), E (Ethylacetate), M (methanol), W1 (water fraction no. 1) and W2 (water fraction no. 2)] of *C. lemon* developed in CEF (top row), EMW (middle row) and BEA (bottom row) and sprayed with vanillin (left), 1, 2-diphenylpicryl hydrazyl (middle) and *Mycobacterium smegmatis* (right)

With *A. dimidiata* sub-fractions, constituents on plates eluted in BEA were best resolved with antioxidant constituents being of high polarity. Similar compounds with anti-mycobacterial activity were present in all the sub-fractions although the concentration in the hexane extract was low (Fig. 3). Sub-fractions of *C. heroroense* showed presence of compounds with antioxidant activity that were best resolved in the CEF eluent system and that for anti-mycobacterial activity contained in the ethyl acetate fraction in the EMW eluent system (Fig. 4). *C. lemon* on the other hand, did not show antimycobacterial activity with the sub-fractions (Fig. 5).

### Minimum inhibitory concentration of sub-fractions against *M.smegmatis*

Table 5 represents MIC values obtained for sub-fractions of the selected plants tested against *M.smegmatis*. In general potent to moderate activity was shown for sub-fractions between the ranges of MIC 0.04 to 0.63 mg/ml. The butanol fraction of selected plants showed moderate activity with average MIC value of 0.16 mg/ml followed by the hexane and methanol sub-fraction (0.19 mg/ml). The hexane subfraction of *A.dimidiata* showed the most potent activity (MIC 0.04 mg/ml) as well as moderated activity with the butanol, ethyl acetate, methanol fractions (MIC 0.16 mg/ml).

**Table 5:** Minimum inhibitory concentration (MIC) in mg/ml of the solvent- solvent fractions of *Citrus lemo*n (CL), *Combretum heroroense* (CH) and *Adipodytes dimidiata* (AD) against *M.smegmatis*

Plant species	But	Hex	EtAc	MeOH	H_2_O no 1	H_2_O no 2
*C.lemon*	0.16	0.24	0.63	0.16	0.31	0.31
*C.heroroense*	0.16	0.31	0.31	0.24	0.16	0.63
*A.dimidiata*	0.16	0.04	0.16	0.16	1.25	1.25
Average	0.16	0.19	0.37	0.19	0.57	0.73

Rifampicin = 0.13 mg/mL

But (butanol), Hex (hexane), EtAc (ethylacetate), MeOH (methanol), H_2_O no 1 (water) fraction 1 and H_2_O no 2 (water) fraction 2

### Minimum inhibitory concentration using *M. tuberculosis*

The plant with the highest average total activity was *A. dimidiata*. Based on the bioautography results and MIC results of sub-fractions, the hexane and butanol fractions of A. dimidiata were further tested for activity against tuberculosis (TB) strain and multi- drug resistance (MDR) - field strain of TB. The table below (table 6) shows the activity of the butanol and hexane fractions against the MDR field strain and the H37Rv TB strain. The extracts showed good activity against the MDR field and H37Rv strains with MIC values of 0.47 mg/ml and 0.31 mg/ml respectively. The extracts show more potent activity against MDR field strains than the positive controls (isoniazid, rifampicin, streptomycin).

**Table 6:** Minimum inhibitory concentration (mg/ml), values of the butanol and hexane sub-fractions of *A. dimidiata* (AD) against MDR field strain and H37Rv strain of *M. tuberculosis*

Fraction/control	MDR field strain	H37RV (ATCC)
AD Butanol	0.47	0.31
AD Hexane	0.47	0.31
Isoniazid (INH)	>2.5	<0.02
Rifampicin	>2.5	<0.08
Streptomycin	>2.5	<0.02
Acetone	>2.5	>2.5

## Discussion

The quest for development of new drugs amidst an ever growing multiplicity of infectious pathogens is high. The trend is further complicated by the development of resistance by pathogens posing a threat to the wellbeing of mankind. In this study, extracts and sub-fractions of *C. lemon*, *C. heroroense* and *A. dimidiate* were evaluated for their antimycobacterial activity against *M. smegmatis* and the most active fractions tested against MDR field strain of TB.

Prior to testing, selected plants were each extracted using hexane, dichloromethane, acetone and methanol. Methanol was the best extractant with an average percentage yield of 4.16 for the three plants while hexane had the least. The highest percentage yield of methanol is related to the presence of more polar constituents within the plants. It is therefore not surprising that traditional healers use mostly water for extraction processes. To determine the fingerprint profiles of the extracts, spotted plates on TLC were eluted in solvents of varying polarity. Constituents in all the plants were best separated in the BEA eluent system when compared to those in the more polar eluent system (EMW). The different plants were thus evaluated for the presence of various constituents such as saponins, phlobatannins, tannins, terpenes/terpenoids, steroids,cardiac glycosides and flavanoids. *A. dimidiata*, *C. heroroense* and *C. lemon* were not shown to contain phlobatannins. Similarly cardiac glycosides were not detected in *C. lemon* and *A. dimidiata*, and saponins in *C. heroroense*. Finding in this study on the tested phytochemicals contained in *A. dimidiata* and *C. heroroense* is consistent with previous reports [20]. Although other authors [21] have reported the presence of saponin in the ethanolic extract of peels as well as cardiac glycosides in the pulp and peels of the water and ethanolic extracts of *C. lemom*, its presence was not detected in the acetone leaf extract in this study. A possible explanation for this variation could be the difference in concentration of these phytochemical in the different plant part, as have been observed in other studies evaluating phytochemical constituents in different plant parts [22].

The anti-mycobacterial activities of crude extracts of the hexane, dichloromethane, acetone and methanol extracts of each of the selected plant species were then assessed by determination of their MIC values against *M. smegmatis*. In general all extracts of the selected plant exhibited some form of activity against the tested pathogen. The dichloromethane extract of *A. dimidiata* was shown to possess potent anti-mycobacterial activity with MIC value of 0.1 mg/ml, followed by the dichloromethane and methanol extracts of *C. lemon* (MIC 0.3 mg/ml) while other extracts of the different plant species had minimal activity. Total activity values were equally shown to be higher for the dichloromethane of *A. dimidiata* and those of the methanol extracts of *C. lemon* and *A. dimidiata*. Foubert et al., [23], isolated six compounds from *A. dimidiata* namely apodytine (A-F) which were found not to be active against *S. aureus* and *E. coli* at concentrations tested. Furthermore, minimal antibacterial activity going by MIC values have also been recorded for the acetone extract of this plant against *S. aureus*, *E. coli*, *E. faecalis* and *P. aeruginosa* even when bioautographic results show the presence of compound with activity against *E. coli* [24]. On the other hand, available reports [25] also show presence of compounds active against *M. smegmatis* in *A. dimidiata* and *C. hereroense* extracts on bioautography even when minimal activity on MIC was recorded for these plants. The presence of anti-mycobacterial constituents in *C. lemon* is consistent with previous reports [26] where it has been shown to exhibit strong anti-mycobacterial activity against *M. smegmatis*.

The growing interest in combination therapy or polypharmacy as against the “silver bullet” approach to achieve therapeutic benefits for a number of diseases prompted us to investigate the combined effect of the plant extracts against *M. smegmatis*. Constituents in medicinal plants are complex in nature, implying their ability to exert their therapeutic effect either in consonance, as single entities or can antagonise the therapeutic effect of an otherwise active component. Since traditional herbal preparations most at times comprise of more than one plant, the effect of the combination of different extracts of the same and those of extracts of the different plants was assessed for possible synergistic effects. This implies that the combined action of constituents within the extracts when taken together can increase each other's effectiveness. The phenomenon of synergistic effects is often crucial to bioactivity in plant extracts and activity may be lost in some cases, in purified fractions.

In this study, minimum inhibitory concentration values of up to 0.16 mg/mℓ were considered to reflect good anti-mycobacteria activity against *M. smegmatis*. The combination of the hexane and acetone, and the dichloromethane and methanol extracts of *C. heroroense* and *A. dimidiata* exhibited a more potent activity (MIC 0.04 mg/ml) than either of the extract of the plants alone, followed by the acetone and methanol, and hexane and methanol extracts combinations (MIC 0.08 mg/ml). A similar activity (MIC 0.08 mg/ml) was also obtained for the combination of the dichloromethane and acetone extracts of *C. lemon* and *C. heroroense*. With same extracts of the different plants, potent activity was also shown for the acetone-acetone and dichloromethane- dichloromethane extracts of *C. heroroense* and *A. dimidiata* (MIC 0.08 mg/ml). The observed enhances activities may be related to a possible potentiation or synergistic effect of components present in the extracts of the different plants. Such beneficial effects in herb-herb combination and its scientific relevance have been well documented [27].

The plants were further sub-fractionated into the hexane, butanol, ethyl acetate, methanol, water fraction no. 1 and water fraction no. 2 and assessed for the presence of antioxidant activity against *M. smegmatis* on TLC. All the fractions of the three plants namely *A. dimidiata*, *C. heroroense* and *C. lemon* contained constituents with different colours indicating the presence of variety of compounds. Sub-fractions of *A. dimidiata*, were best resolved in BEA with antioxidant constituents being of high polarity. All the sub-fractions contain compounds with anti-mycobacterial activity, although the concentration in the hexane extract was low. Sub-fractions of *C. heroroense* showed presence of compounds with antioxidant activity that were best resolved in the CEF eluent system and that for anti-mycobacterial activity contained in the ethyl acetate fraction in the EMW eluent system. *C. lemon* on the other hand, did not show anti-mycobacterial activity with the sub-fractions suggestive of a synergistic effect of compounds contained in the crude extract [26] with possible loss of activity through sub-fractionation. Compounds that exhibited anti-mycobacterial activity on bio-autograph were not shown to correspond to those with antioxidant activity. It can therefore be said with some degree of certainty that the compounds contained in the fractions with antioxidant activity when acting alone are not responsible for the observed anti-mycobacterial effects recorded in this study. The anti-mycobacterial activity observed in the bioautograms will assist in bioassay guided isolation of pure compounds.

In general, apart from the butanol fraction of all the plants and hexane fraction of *A. dimidiata*, sub-fractionation of the crude plants, were not shown to markedly result in a more potentiated activity going by MIC values when compared to the crude itself. This finding was not surprising, since recent report have shown fractionation to reduce cytotoxicity rather than enhance the anti-helminthic activity of *Heteromorpha arborescens* [28]. The butanol sub-fractions of selected plants showed moderate activity with average MIC value of 0.16 mg/ml followed by the hexane and methanol sub-fraction (0.19 mg/ml). The hexane sub-fraction of *A. dimidiata* showed the most potent activity (MIC 0.04 mg/ml), followed by the butanol, ethyl acetate and methanol fractions (MIC 0.16 mg/ml) against *M. smegmatis*.

Based on total activity values, bio-autography and MIC results of sub-fractions, the hexane and butanol fractions of *A. dimidiata* were further tested for activity against a tuberculosis strain and multi- drug resistance (MDR) - field strain of TB. The butanol and hexane sub-fractions of *A. dimidiate* were shown to exhibit potent activity against the MDR field strain with MIC values of 0.47 mg/ml and 0.31 mg/ml respectively, greater than the positive controls (isoniazid, rifampicin, streptomycin). The inner compartment of mycobacteria, consists of peptidoglycan (PG), arabinogalactan (AG), and mycolic acids (MA) covalently linked together to form a complex known as the MA-AG-PG complex that extends from the plasma membrane outward in layers. Many of the drugs used to combat mycobacteria target the MA-AG-PG complex. It is thus likely that based on polarity the hexane and butanol sub-fractions contain constituents that work through a similar mechanism.

## Conclusion

The plant species *C. lemon*, *C. heroroense* and *A. dimidiata* all possess compounds with antioxidant activity, suggestive of their relevance in the scavenging of free radicals that may build up in a disease condition. All the extracts of plants tested exhibited some degree of anti-mycobacteria activity which were more enhanced when extracts of the same plant or combination of plants in the study were employed. Sub-fraction in some cases can reduce activity of extracts especially where the desired effect is synergy dependent. The hexane and butanol sub-fractions of *A. dimidiata* exhibited potent anti-mycobacteria activity more that the positive control against the MDR-field strain. Studies are ongoing with the aim of assessing the cytotoxic effects of these fractions in-vitro and isolation of compounds within the fractions that are responsible for the observed activity. The result of this finding is significant, considering the increase in the number of MDR-TB affected countries, and the need for development of more potent anti-mycobacteria drugs against drug resistant strains of the pathogen.

## Abbreviations

ATCC: American Type Culture Collection
BEA: Benzene: ethanol: ammonia hydroxide
CEF: Chloroform: ethyl acetate: formic acid
EMW: Ethyl acetate: methanol: water
MDR-TB: Multidrug resistant *Mycobacterium tuberculosis*
TB: Tuberculosis
TLC: Thin layer chromatography
